# Interpretation of the Recombination Lifetime in Halide Perovskite Devices by Correlated Techniques

**DOI:** 10.1021/acs.jpclett.2c01776

**Published:** 2022-08-03

**Authors:** Juan Bisquert

**Affiliations:** Institute of Advanced Materials (INAM), Universitat Jaume I, 12006Castelló, Spain; Yonsei Frontier Lab, Yonsei University, Seoul03722, South Korea

## Abstract

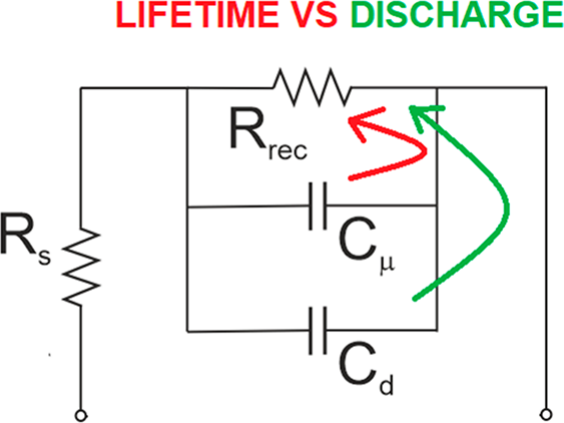

The recombination lifetime is a central quantity of optoelectronic devices, as it controls properties such as the open-circuit voltage and light emission rates. Recently, the lifetime properties of halide perovskite devices have been measured over a wide range of the photovoltage, using techniques associated with a steady state by small perturbation methods. It has been remarked that observation of the lifetime is affected by different additional properties of the device, such as multiple trapping effects and capacitive charging. We discuss the meaning of delay factors in the observations of recombination lifetime in halide perovskites. We formulate a general equivalent circuit model that is a basis for the interpretation of all the small perturbation techniques. We discuss the connection of the recombination model to the previous reports of impedance spectroscopy of halide perovskites. Finally, we comment on the correlation properties of the different light-modulated techniques.

The recombination lifetime, τ_rec_, is a central quantity to the analysis of semiconductor optoelectronic devices such as solar cells. τ_rec_ is the time for recombination of injected or photogenerated electrons and holes.^1^ The measured lifetime may be associated with a single microscopic mechanism or correspond to a composition of them, such as band-to-band radiative recombination and Shockley–Read–Hall defect-mediated nonradiative recombination.^2,3^

The electronic carrier lifetimes in a material can be investigated by optical stimulation of a contactless film, which contains only the light absorber material over a substrate, using a range of time-resolved optical techniques, including transient absorption spectroscopy (TAS), optical-pump-terahertz-probe (OPTP), time-resolved-microwave-conductivity (TRMC), time-resolved-photoluminescence (TRPL), time-resolved-2D-Fourier-transformed-infrared spectroscopy (TR-2D-FTIR), and other techniques.

However, one is generally interested in obtaining the lifetimes in working devices with contacts. The optical methods are still useful tools, probably with different results because of the additional properties introduced by the electrodes, such as interface-induced recombination. In addition, for a full device it is possible to measure the electrical quantities of current and voltage, and a new set of methods to determine lifetimes appears. The recombination parameters may be established by purely stationary techniques, such as obtaining the ideality factor of the exponentially raising current dependence on voltage.^4,5^ But a number of time- and frequency-resolved methods provide more direct information on the required kinetic parameters.

There are two types of such “dynamic” methods. We may establish a large perturbation and observe the decay to equilibrium, as in the open-circuit voltage, *V*_oc_, decay (OCVD) that provides the lifetime over a wide voltage range with a single measurement.^6,7^ Sometimes large signal techniques are the method of choice, as in the measurement of the current–voltage curve. It is also beneficial that with one measurement you can get information on different mechanisms. However, the parameters change strongly in the course of the measurement when the lifetime is concentration- or voltage-dependent, providing some uncertainties of interpretation. We aim to obtain parameters that can be assigned to a given situation and measured by different techniques to show the coherence of the methods.

The procedure that achieves this goal is to use a “differential” or “small perturbation” method. Here, the system is fixed at a steady state that is not affected by the perturbation associated with the measurement. Furthermore, in this type of measurement one can apply a perturbation either in time, *i.e.*, a step variation or a short square pulse, or in frequency domain, by a small oscillation perturbation with a certain angular frequency ω. A range of methods appears. The small perturbation techniques in the time domain are^8^

- TPV, transient photovoltage: records the dynamics of the *V*_oc_ drop of a cell after it has been exposed to a short illumination pulse;
- TPC, transient photocurrent: records the dynamics of the current drop after a laser pulse.

The small perturbation methods in the frequency domain are^9^

- IS, impedance spectroscopy: gives the transfer function *Z* of a sinusoidal voltage with respect to a sinusoidal current;^10^
- IMPS, intensity-modulated photocurrent spectroscopy: gives the transfer function *Q* of a sinusoidal current with respect to a sinusoidal illumination flux;
- IMVS, intensity-modulated photovoltage spectroscopy: gives the transfer function *W* of a sinusoidal voltage with respect to a sinusoidal illumination flux.

In principle, the time domain and frequency domain methods, if made over an identical set of steady-state conditions, must correspond to each other by the Laplace transformation. The correspondence is more or less complex depending on the properties of the system.^8^ If a given device is fully controlled by recombination, the lifetime is not difficult to measure, as the time transient or frequency spectra can be interpreted unambiguously. But often, real devices are composed of different relaxation processes, associated with combination of transport, recombination, inhomogeneities, defect accumulations, interfaces, and so on. Then multiple time constants or complex spectra are obtained, with more or less distinct features that need methods of interpretation to reach the recombination lifetime, separated from other ongoing processes.^11^ The use of an equivalent circuit is substantial to the frequency techniques and provides an excellent framework to specify the models being used in each technique or approach and to achieve the separation of the existing dynamic components.^9^

There is, therefore, a significant opportunity to reconcile different methods to obtain a robust set of system parameters. In the studies of previous hybrid photovoltaic technologies it was established that analyzing the mechanisms of the lifetime requires obtaining high-quality data over a wide variation of the splitting of the Fermi level.^12,13^ Recombination in halide perovskites has been studied for many years, but recently some works have developed the correspondence of optical and electro-optical techniques over a wide voltage range.^14−16^ In addition, progress has been obtained in the characterization of capacitances of halide perovskites.^9,17,18^ Herein, we summarize the progress in this topic by formulating the conditions of observation of the lifetime in halide perovskites in terms of the capacitances in the system, and we show the correspondence of the models used in the time domain with the frequency domain methods. We also summarize the problems still existing for the measurement of the lifetime in order to point out further experimental determinations.

As explained before, herein we discuss the differential or small perturbation recombination lifetime τ_rec_. The general concept has been explained in a textbook.^1^ τ_rec_ is associated with a specific recombination mechanism as defined later in eq 7. There are other possible meanings of a lifetime: it can be a fit to a large signal or small signal observable (voltage, current, luminescence, conductivity, *etc*.). This will be denoted a “response time”, associated with a given measurement.

The relation of the recombination lifetime to the response time^19^ is a general problem that appears when applying the small perturbation methods mentioned above to measure the lifetime. When the measurement is affected by additional factors related to electronic dynamics in the sample, such as capacitive charging, or trapping and detrapping effects, that occur prior to recombination, one obtains an “effective recombination time”, τ_eff_, that is a response time. In many cases we have the decomposition
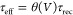
1where θ is a factor that depends strongly on the difference of the Fermi levels of electrons and holes (expressed as a voltage *V*). Typically, θ ≫ 1 indicating the additional time of the processes that slow down the measurement, composed with the fundamental electron–hole recombination events that take a time τ_rec_.

In order to obtain the fundamental lifetime that can be observed by small perturbation techniques, it is necessary to identify the nonrecombinative effects that are included in θ(*V*). These effects have been well-recognized in past technologies such as the silicon solar cell (by the depletion capacitance),^10,14,20^ organic solar cells (by capacitive charging),^21,22^ and the dye-sensitized solar cell (by multiple trapping effects).^23,24^

For example, for the case of trap-limited recombination, the factor is given by the variation of localized (*n*_L_) to free carriers (*n*_c_) in the conduction band:^24,25^
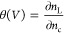
2The factor in eq 2 is valid in a quasistatic approximation, when trapping and detrapping is fast, for a small perturbation measurement.^23,26^ This is because during measurement of the recombination lifetime you need to free a slice of localized charge when the Fermi level is displaced, as pointed out by Rose.^19^ For an exponential tail of localized states, eq 2 depends exponentially on voltage, so that exponential dependence of the lifetime observed in experiments may not be a property of the recombination mechanism.^27^ In the multiple trapping model a similar effect exists for the diffusion coefficient,^28^ and the corresponding factor is given by (*∂n*_L_/*∂n*_c_)^−1^. It turns out that the measured diffusion length *L*_eff_ = (*D*_eff_τ_eff_)^1/2^ becomes independent of trapping factors.^23,24,26^

A summary of the properties of the measured electron lifetimes in halide perovskites is presented in Figure 1.^15^ There are exponentially decreasing regions at increasing electron density and also some regions of constant lifetime. Another important feature is shown in Figure 2. The lifetime obtained by TPV is consistently larger than that measured by optical methods. It has been pointed out that some exponential dependencies and excess values are due to capacitive factors.^14−16,29^ We now analyze the recombination model used in these references in order to obtain the excess factor of eq 1.

**Figure 1:**
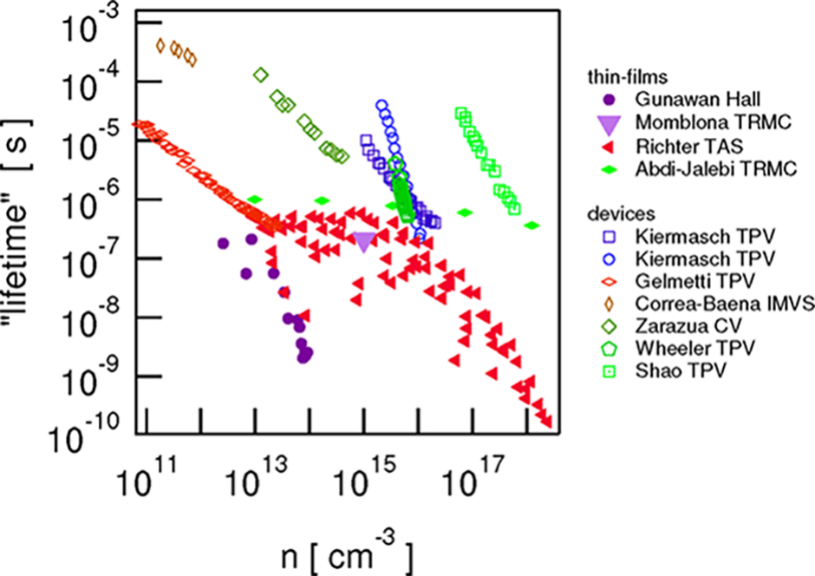
Reported carrier lifetimes (derived from different techniques) versus carrier densities for thin-films (solid symbols) and devices (open symbols). Reproduced with permission from ref (15). Copyright 2021 The Authors of ref (15).

**Figure 2:**
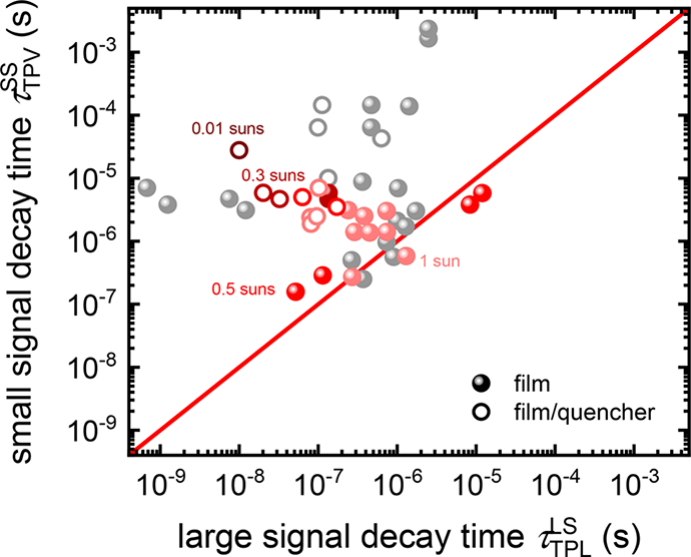
Data collection from literature, comparing the decay time τ_LS,TPL_ of the transient PL measured on perovskite films (solid symbols) or on perovskite/transport layer stacks (open symbols) with the stated decay time τ_SS,TPV_ resulting from transient photovoltage measurements on the respective solar cell device. The color code is linked to the bias light intensity during the TPV experiment ranging from light red (1 sun) to dark red (0.01 suns). For the gray data points, no information about the bias illumination level was available. The bisecting line (red) serves as a guide to the eye and indicates where both decay times are equal. The comparison of these decay times highlights that they correlate poorly and can differ by orders of magnitude. Interestingly, the decay time constant from the TPV measurement is usually longer, although recombination losses are expected to be higher in the complete solar cell than in the pure perovskite film. Reproduced with permission from ref (16). Copyright 2021 The Authors of ref (16).

Let *n* be the electron density. We assume that traps are saturated so that *n* represents free carriers. Their density depends on the voltage *V* as
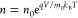
3Here, *q* is the elementary charge, *k*_B_ Boltzmann’s constant, *T* the absolute temperature, and *m*_0_ an ideality factor. If electrons are minority carriers, then *m*_0_ = 1, while at high fluence *n* = *p* we have *m*_0_ = 2. For illustration of the methods we will assume this last value in the simulations, which is connected to measurement of lifetimes at high irradiation densities, although the actual values in high-performance solar cells may be different.^30^

In a dynamic situation, the variation of carrier density is
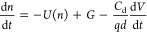
4The recombination rate, *U*, and the generation rate, *G*, are both in cm^3^ s^–1^. Equation 4 implies that the carrier density in the steady state (indicated by the overbar) is given by the solution of
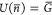
5The first three terms in eq 4 are volumetric, and they are supposed to be the same throughout the film, so the system must be in homogeneous conditions such as the open-circuit. The last term of eq 4 accounts for capacitive charging of the electrodes separated a distance *d*, the thickness of the active film. The dielectric capacitance *C*_d_ is given in F cm^–2^. The meaning of *C*_d_ is explained in more detail later. The factor 1/*d* converts the surface charge to volumetric charge.

We can prepare a homogeneous steady state and produce a small perturbation of the form *n* = *n̅* + *n̂*(*t*), (with *n̂* ≪ *n̅*), *V* = *V̅* + *V̂*(*t*), *etc*. Consider the decay of a small perturbation of charge in the dark, for a sample without contacts. Equation 4 gives
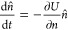
6Whatever the form of *U*(*n*), the decay is exponential, and the differential recombination lifetime is given by^24^
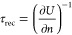
7To make the meaning clear we adopt the recombination model that has been observed in many measurements of halide perovskites.^4,15,16,31−33^
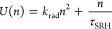
8Here *k*_rad_ is the coefficient for radiative band-to-band recombination and τ_SRH_ is the lifetime for linear trap-assisted Shockley–Read–Hall recombination. Inclusion of Auger recombination is not normally necessary in the framework of the methods discussed here. From eq 7 we obtain the total recombination lifetime as the sum of the two parallel pathways
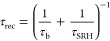
9where
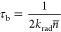
10If we apply a small perturbation of voltage or incident light, the systems respond according to the equation obtained from eq 4
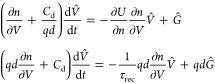
11Let us introduce some convenient quantities. The chemical capacitance is^34^
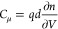
12The factor *d* makes *C*_μ_ expressed in F cm^–2^. The generation flux Φ_g_ (in cm^–2^) is
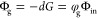
13where φ_g_ is a generation efficiency and Φ_in_ is the incoming light flux per unit area (cm^–2^). Equation 11 can be presented as
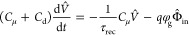
14Suppose that Φ̂_in_ = 0 during the measurement. Then, from eq 14
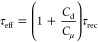
15We reach the conclusion that the lifetime is *directly* measured only if the chemical capacitance is larger than the dielectric capacitance. In the domain in which *C*_d_ > *C*_μ_, the dominant process is a discharge of the dielectric capacitance. The effective lifetime is larger than the recombination lifetime, as indicated in the observations of Figure 2, by the factor θ(*V*) = *C*_d_/*C*_μ_.

In the model of eq 14 we have generally termed “dielectric capacitance” any capacitance that responds to the electrical field, either in very short-range, as the surface ionic polarization, or in long-range, as the bulk dielectric response. In an optoelectronic semiconductor device, *C*_d_ can have different origins:^18^ (a) The metal contacts contribute a constant capacitance, affected by additional specific capacitances due to compact or passivating layers,^35^ and in the presence of mobile ions the contacts include a Helmholtz capacitance. (b) A semiconductor depletion capacitance is voltage-dependent as characterized in Mott–Schottky plots. (c) The bulk dielectric response (the geometric capacitance), can also be considered constant as a function of voltage. All these terms are included into the dielectric capacitance *C*_d_ in a broad sense.

On the other hand the chemical capacitance is due to the increase of the chemical potential and does not consider electrical field dependence. This distinction is expressed in a textbook.^1^ According to eq 12, *C*_μ_ increases exponentially with the photovoltage as
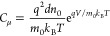
16The chemical capacitance can be measured directly in silicon^20,36,37^ and organic devices.^38−40^ It has the same ideality factor *m*_0_ as that of the carrier density (eq 3).

Consider eq 14 in the dark and with negligible recombination. Then a voltage transient produces the result *C*_μ_ = −*C*_d_ that seems to be a charge compensation equation to satisfy electroneutrality. But in the simple model of eq 14, the chemical capacitance and dielectric capacitance are not connected by charge neutrality.^1^ In order to obtain strict charge compensation one needs to establish a complete semiconductor model of the device (involving Poisson equation, *etc*.).^41^ The model of eq 14 must be taken as a first approximation to the capacitive response that only indicates which type of capacitance is dominant for the measurement of the dynamic properties.

Let us review some results from the literature that consider eq 15. Figure 3 shows a silicon solar cell measured by Mora-Seró *et al.*(10) In Figure 3a,b it is clearly observed that the dominant capacitances in the cell are a depletion capacitance at low voltage and the chemical capacitance at high voltage. The transition occurs at approximately 0.4 V. Above this value the lifetime is a constant (Figure 3c), but below 0.4 V, the *RC* product is affected by the large dielectric capacitance and the result is not a recombination lifetime.

**Figure 3:**
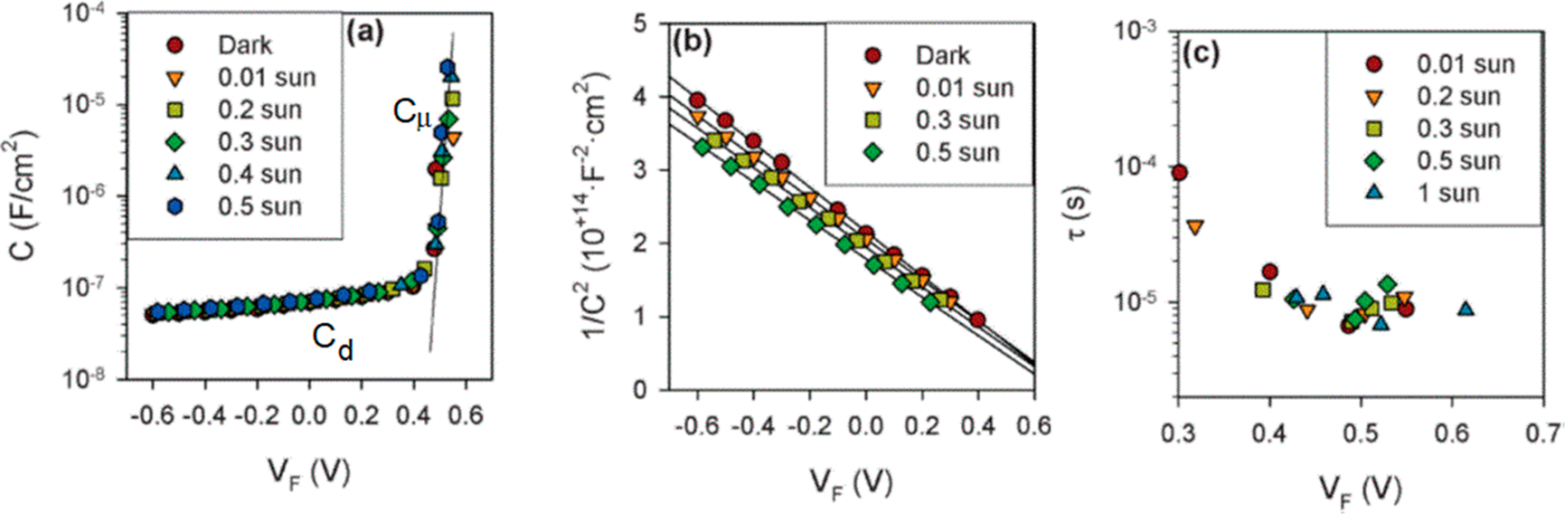
Electrical response of a monocrystalline silicon solar cell. (a) Device capacitance as a function of Fermi-level potential, *V*_F_, for different light intensities. Solid line represents the fit of *C* in the potential region where the chemical capacitance *C*_μ_ is dominant. The depletion capacitance *C*_d_ that is the dominant contribution to the cell capacitance at low voltage is represented separately in panel b as a Mott–Schottky plot. (c) Minority carrier lifetime as a function of Fermi level potential obtained as the product of the recombination resistance and total capacitance. Reproduced with permission from ref (10). Copyright 2009 The Royal Society of Chemistry.

Kiermasch *et al.*(14) also showed the lifetime for a silicon device (Figure 4). For a constant dielectric capacitance we have
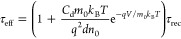
17Thus, the effective lifetime decreases exponentially until the chemical capacitance becomes large enough and reveals the constant recombination lifetime.

**Figure 4:**
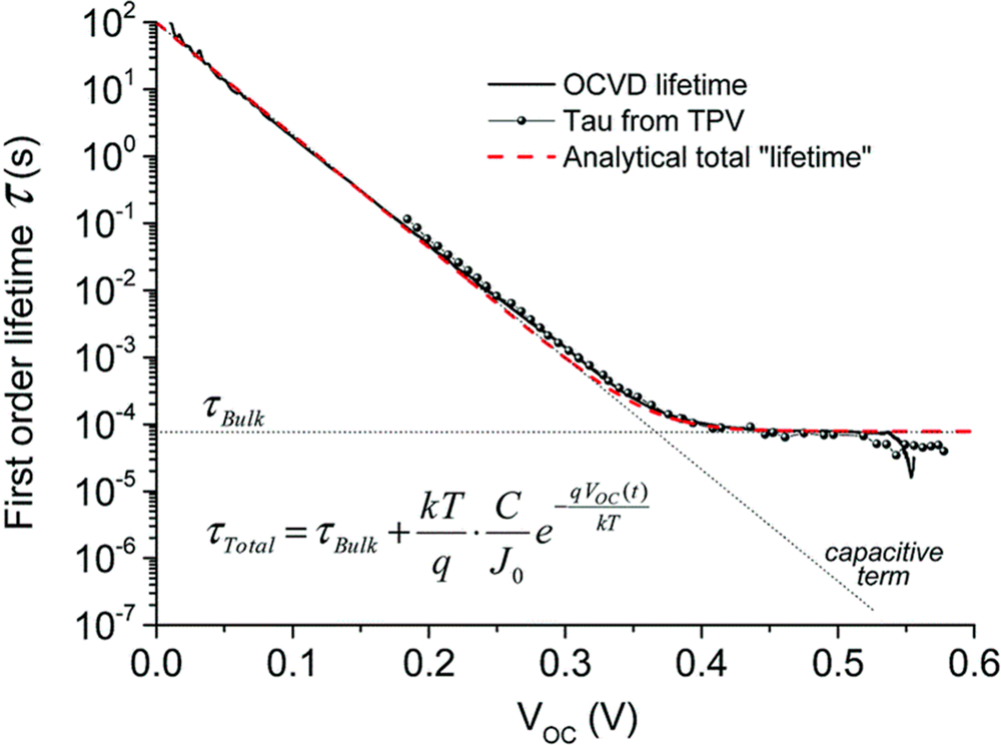
Calculated effective differential lifetime for a Si photodiode together with measured ones from OCVD and TPV. The capacitance value used is 2 × 10^–4^ F m^–2^. Reproduced with permission from ref (14). Copyright 2018 The Royal Society of Chemistry.

Finally, Wheeler *et al.*(42) present in Figure 5a,b the results of differential charging. The chemical capacitance predominates clearly over the substrate capacitance at 0.6 V. As a result, the carrier density can be measured above 0.6 V and the corresponding TPV time constant in Figure 5d gives directly the recombination time.

**Figure 5:**
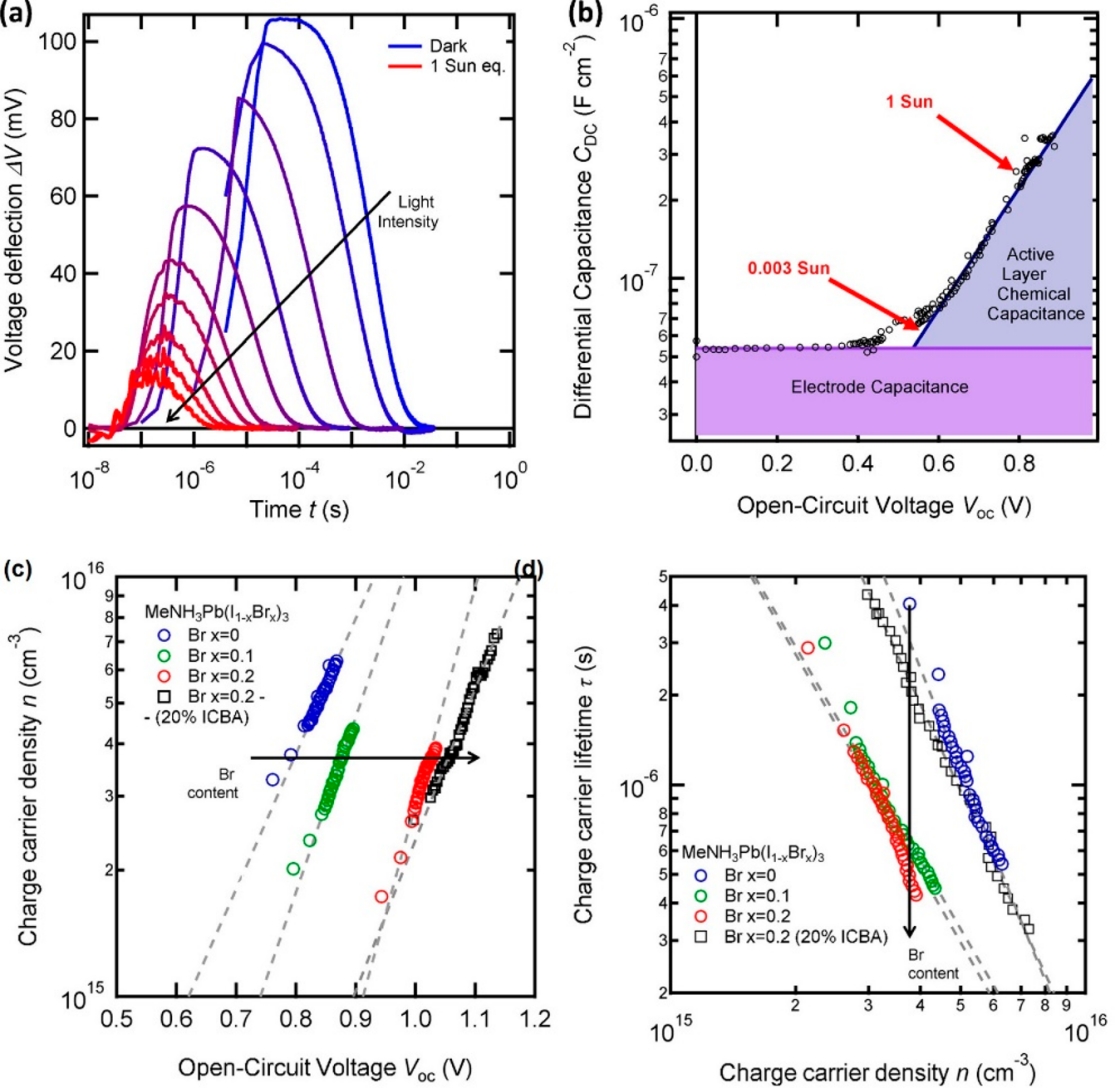
Transient voltage and capacitance response of halide perovskite solar cells: (a) Decay of the transient photovoltage over a range of light intensities between darkness and 1 sun for a CH_3_NH_3_PbI_3_ planar hybrid perovskite. (b) Differential capacitance measured from transient photocurrent and transient photovoltage as a function of the open-circuit voltage (*V*_oc_) over a range of background light intensities from 0 to 5 sun equivalents. (c) Active-layer charge-carrier density (*n*) as a function of quasi-Fermi-level splitting (*V*_oc_). (d) Average charge-carrier lifetime (τ) as a function of *n* for CH_3_NH_3_Pb(I_1–*x*_Br_*x*_)_3_ cells with Br content of *x* = 0, 0.1, and 0.2. The same approximate range of light intensities, between 0.1 and 3 sun equivalents, was used for each material system. The results for the use of 20% ICBA in PCBM for the *x* = 0.2 device is shown with black squares. Reproduced from ref (42). Copyright 2017 American Chemical Society.

We now consider the application of the effective and recombination lifetime to the measurement of halide perovskite solar cells. By eqs 9 and 17, we obtain
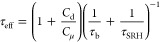
18Normally the radiative lifetime τ_b_ that decreases at increasing voltage becomes dominant (smaller) at high fluence or carrier density. With respect to eq 17 we use a more general form of the dielectric capacitance because it is often observed to depend exponentially on the voltage, as shown in Figure 6.^9^ This topic will be further discussed later, and we assume
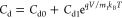
19*C*_d0_ and *C*_d1_ are constants, and *m*_1_ is the ideality factor of the capacitance. *C*_d0_ is a capacitance related to surface polarization. In the simulations, distances are in cm, time in s, voltages in V, charge in C, capacitance in F. For a general illustration of eqs 18 and 19, Figure 7a shows the interplay of capacitances and Figure 7c shows the effective (red) and true (blue) recombination lifetime as well as their different components. We note the transition of recombination mechanisms in the blue line, but both are distorted in τ_eff_ by the capacitive factor, until when *C*_μ_ > *C*_d_, they match each other, τ_rec_ = τ_eff_, as explained earlier. By eq 3 the carrier density is exponential with the voltage, Figures 5c and 7b, so that one can represent the lifetimes either versus voltage or carrier density without changing the form.

**Figure 6:**
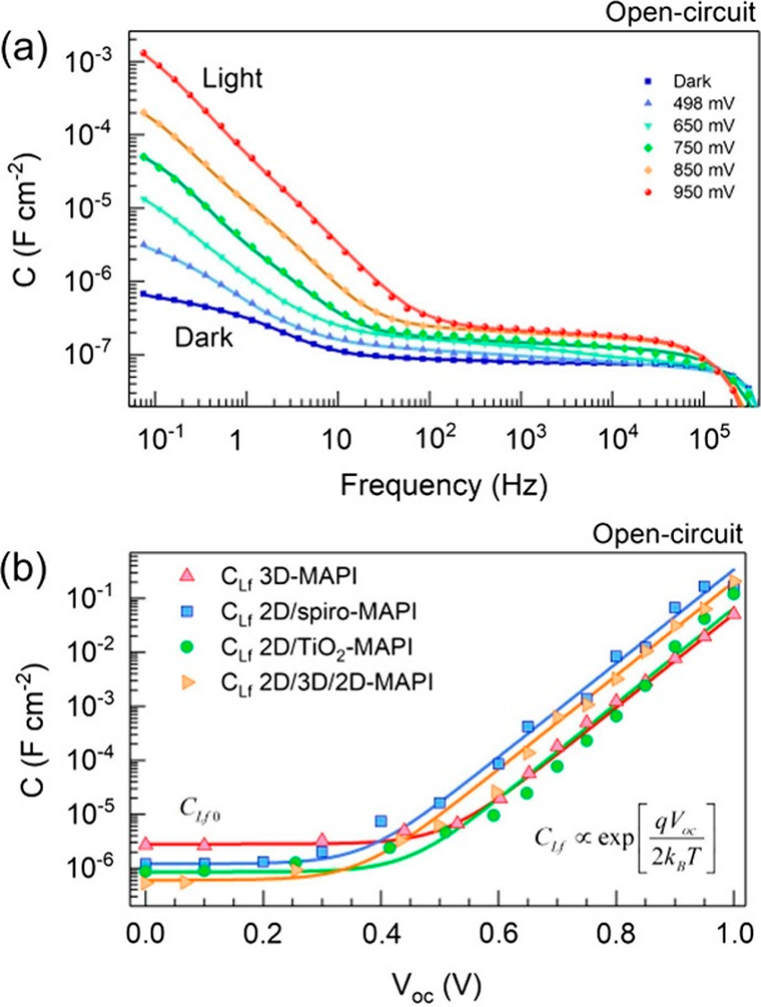
(a) Capacitance spectra as a function of frequency for a CH_3_NH_3_PbI_3_-based perovskite solar cell. (b) Low-frequency capacitance for several devices based on CH_3_NH_3_PbI_3_ and a variety of interlayers (2D perovskite thin capping) also as a function of *V*_oc_. Reproduced with permission from ref (43). Copyright 2019 Elsevier.

**Figure 7:**
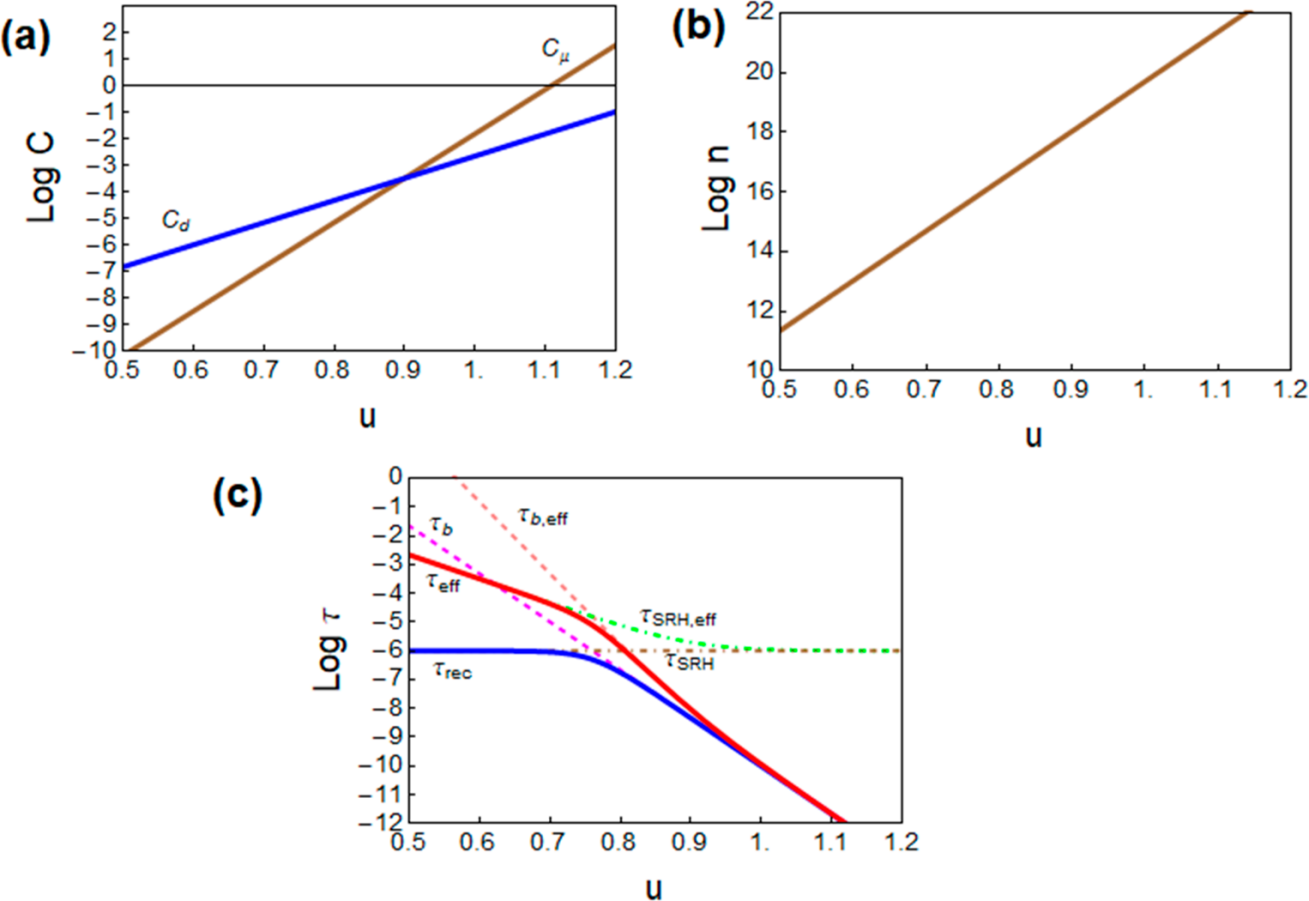
Simulations of (a) dielectric and chemical capacitances, (b) carrier density, and (c) lifetimes as a function of voltage (separation of Fermi levels). *u* is the voltage excluding the drop in the series resistance. Shown in panel c are the recombination lifetime (τ_rec_, blue), the measured lifetime (τ_eff_, red), and their different components in dashed and dot–dashed lines. Parameters: *C*_d0_ = 0; *C*_d1_ = 10^–11^ F cm^–2^; *m*_0_ = 1; *m*_1_ = 2; *k*_rec_ = 1.0 × 10^–10^ cm^3^ s^–1^; τ_SRH_ = 1.0 × 10^–6^ s; *d* = 500 × 10^–7^ cm; *q* = 1.6 × 10^–19^ C; *n*_i_ = 1 × 10^3^ cm^–3^; *k*_B_*T* = 0.026 eV.

Considering the general model (eq 18), we discuss specific results about halide perovskites given in the literature. The data in Figure 8 by Krückemeier *et al.*(16) are highly significant as it compares the film with and without contacts, to identify the effect of the electrodes over a wide range of voltages. They show large-signal TPL measurements of a perovskite film (gray spheres) and the solar cell (red spheres) and TPV measurements (stars). The downward displacement of the TPV data that includes nonradiative recombination, with respect to the gray data of the contactless samples, is due to the combination of two electrode effects: appearance of significant linear recombination and the capacitive effect, as outlined for the same parameters in Figure 9a,b, in which a constant *C*_d_ is adopted.

**Figure 8:**
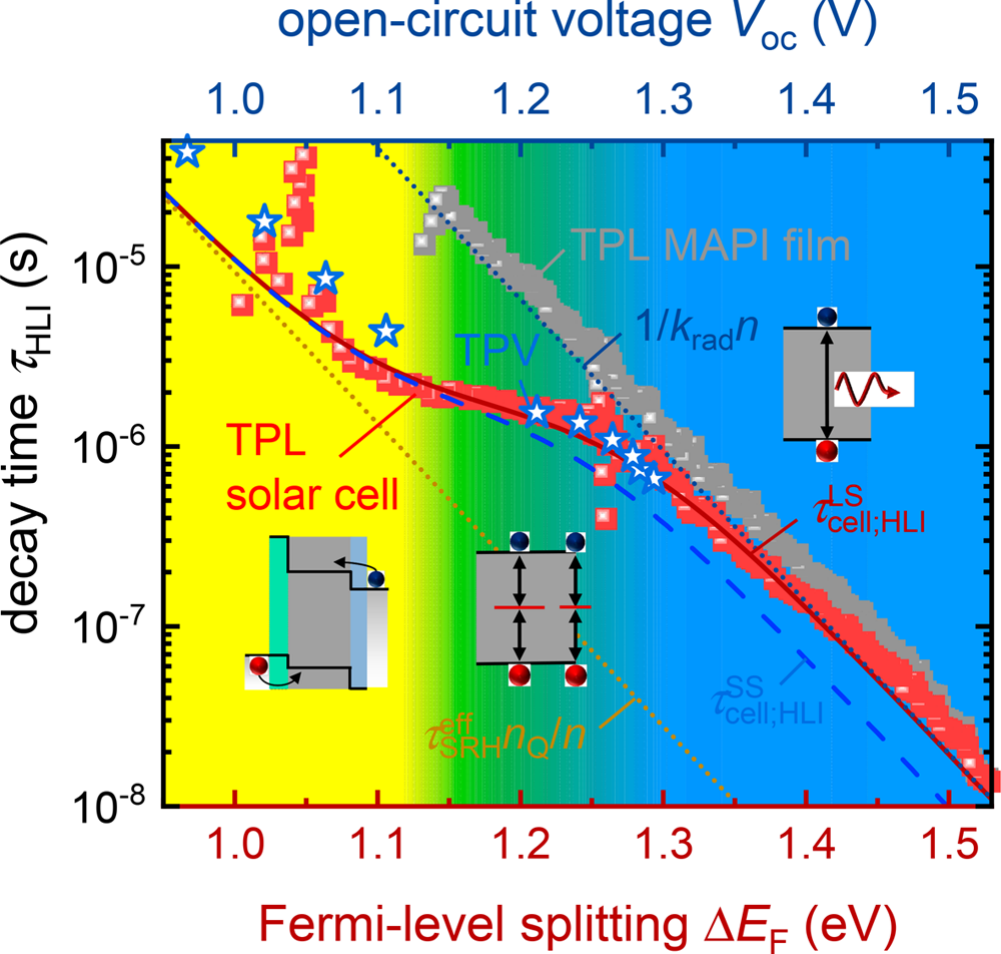
Experimental data of the decay time derived from large-signal TPL measurements (τ_LS,TPL;HLI_) of a perovskite film (gray spheres) and the solar cell (red spheres) and TPV measurements (τ_SS,TPV;HLI_) at different bias illumination intensities (blue stars). Furthermore, the exponential slope of the capacitance-dominated (light yellow) and the radiatively dominated (light blue) regions are shown as a guide to the eye. An analytical model is indicated for small- (blue dashed line) and large-signal (red solid line) transients on cells. Reproduced with permission from ref (16). Copyright The Authors of ref (16).

**Figure 9:**
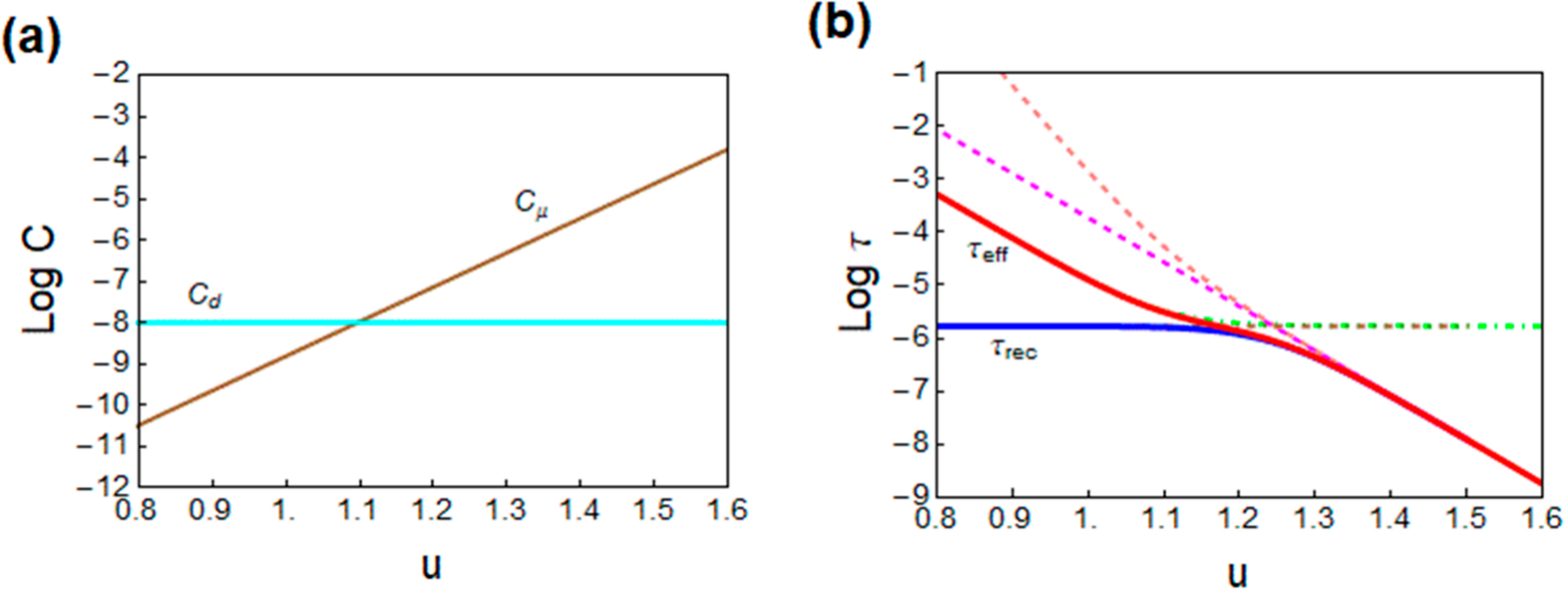
Simulations of (a) dielectric and chemical capacitances and (b) lifetimes. *u* is the voltage excluding the drop in the series resistance. Shown are the recombination lifetime τ_rec_ (blue), the measured lifetime τ_eff_ (red), and their different components in dashed and dot–dashed lines. Parameters taken from ref (16): *C*_d_ = 10^–8^ F cm^–2^; *m*_0_ = 2; *k*_rec_ = 1.5 × 10^–10^ cm^3^ s^–1^; τ_SRH_ = 1.7 × 10^–6^ s; *d* = 280 × 10^–7^ cm; *q* = 1.6 × 10^–19^ C; *n*_i_ = 8 × 10^4^ cm^–3^; *k*_B_*T* = 0.026 eV.

Figure 10 obtained by measurements of TAS by Wolff *et al.* shows the constant lifetime region at low voltage, modified by a small constant capacitive factor, and the transition to the radiative lifetime τ_b_. The different quantities inferred from the experimental parameters are summarized in Figure 11. In Figure 10, the region of constant lifetime is better appreciated than in Figure 8. This is because Figure 10 has a shorter τ_SRH_. The parameter *n*_i_ is very important as it fixes the value of the chemical capacitance. It is taken as the same value in both simulations.

**Figure 10:**
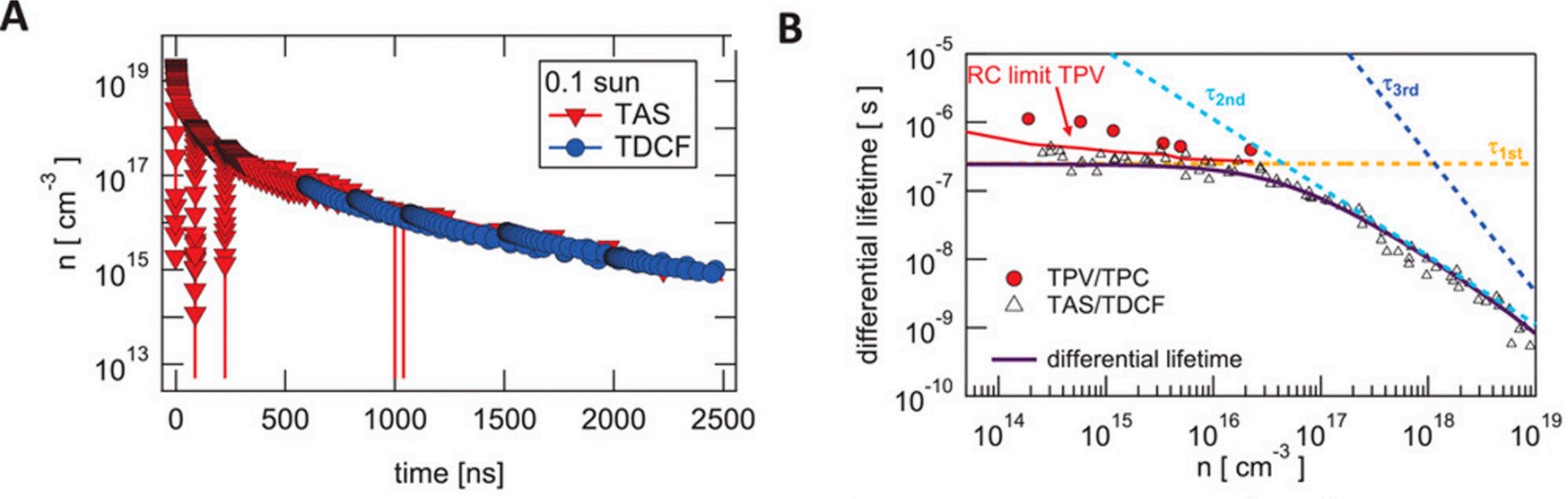
(A) Determination of lifetimes by combined TAS (red) and time-delayed-collection-field (TDCF) (blue) under 0.1 sun equivalent background illumination on full devices at *V*_oc_. (B) Differential lifetimes deduced from transient charge carrier dynamics under 0.1 sun equivalent background illumination and TAS (triangles) and from TPV (red circles) as a function of carrier density. The red line shows the limit for TPV measurements according to Kiermasch *et al.*;^14^ the solid line shows the numerical derivative of the total fit (purple), and the dashed lines show the corresponding contributions as denoted. Reproduced with permission from ref (15). Copyright 2021 The Authors of ref (15).

**Figure 11:**
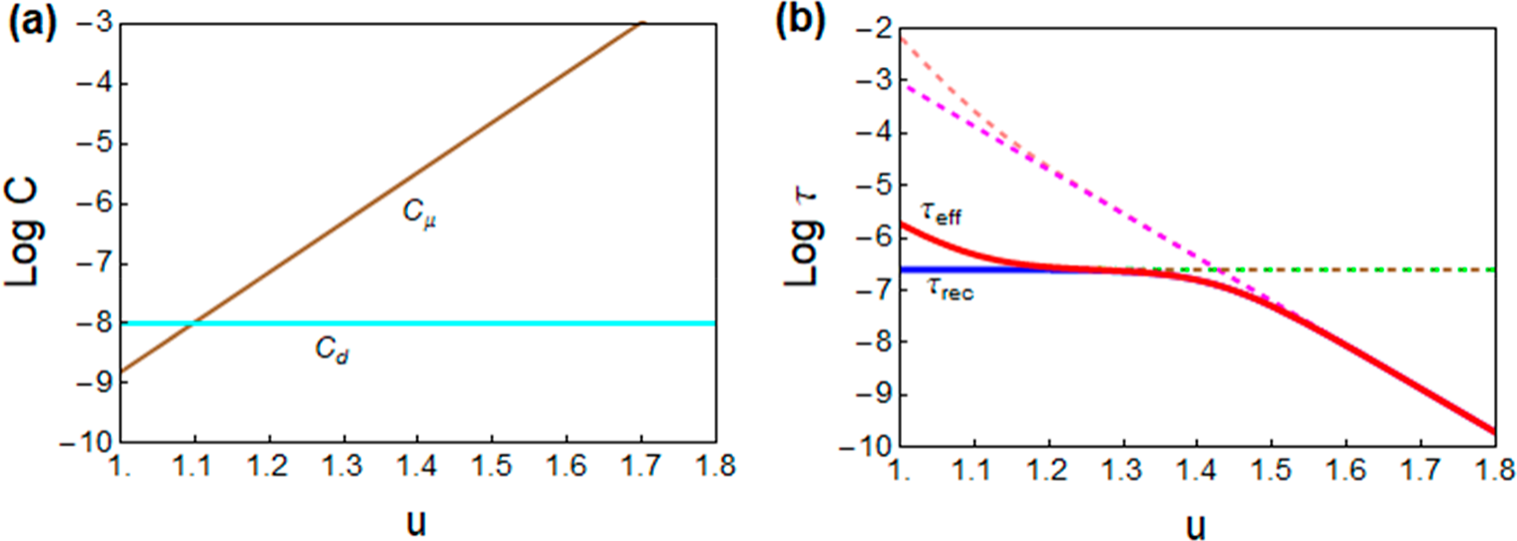
Simulations of (a) dielectric and chemical capacitances and (b) lifetimes. *u* is the voltage excluding the drop in the series resistance. Shown are the recombination lifetime τ_rec_ (blue), the measured lifetime τ_eff_ (red), and their different components in dashed and dot–dashed lines. Parameters taken from ref (15): *C*_d_ = 10^–8^ F cm^–2^; *m*_0_ = 2; *k*_rec_ = 3 × 10^–11^ cm^3^ s^–1^; τ_SRH_ = 0.25 × 10^–6^ s; *d* = 280 × 10^–7^ cm; *q* = 1.6 × 10^–19^ C; *n*_i_ = 8 × 10^4^ cm^–3^; *k*_B_*T* = 0.026 eV.

Incidentally we remark that the large signal measurement and small perturbation methods can be put easily into correspondence if (1) the lifetime is measured separately in the given method and (2) the functional form is a constant or an exponential dependence with voltage^6^ see (Figure 4 and 8). In the case of multiple relaxation phenomena, a more involved method of integration from a set of small perturbation measurements can be used to obtain the response to a large voltage sweep, as in the case of hysteresis in current–voltage curves.^44^ Alternative full drift diffusion numerical simulations can address different methods.^45^

The previous reasoning is based on the time domain decay techniques represented by eq 14. Now we want to develop the electrical analogues of the model in order to apply it to the small perturbation frequency techniques. The recombination resistance is defined as^24^
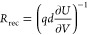
20We can express eq 14 as
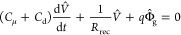
21Clearly this last result consists of the addition of three small perturbation currents: capacitive, recombination, and photogeneration. Furthermore, eq 21 is for the techniques operating at open circuit, which is an assumption of eq 4. If we allow electrical current extraction *I* through the contacts we can write more generally
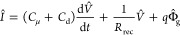
22The correspondent generalization of the large signal eq 4 is
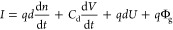
23For the small perturbation measurements at the angular frequency ω we use the Laplace transformation d/d*t* → *iω*. Equation 22 gives
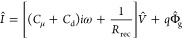
24This last equation can be translated into the equivalent circuit of Figure 12a. *Î* is the current across the series resistance *R*_s_, and the voltage between the external connections is
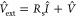
25

**Figure 12:**
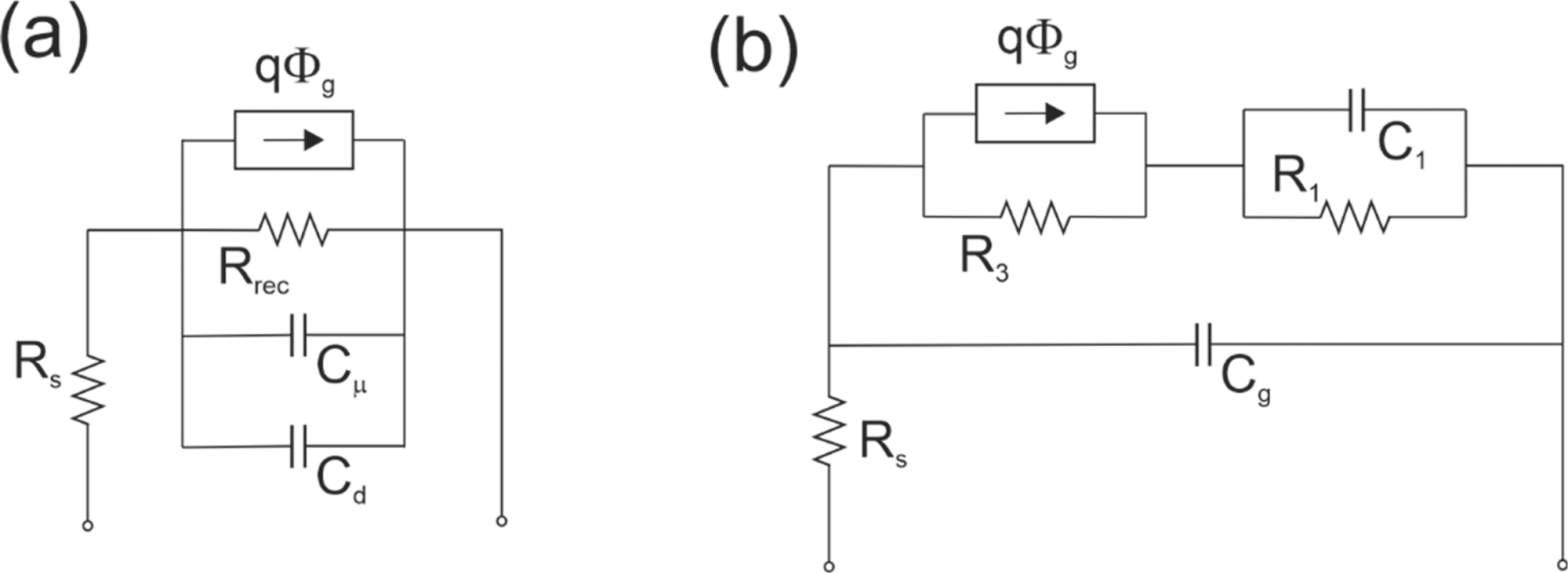
Equivalent circuit representation of small perturbation models. *q*Φ̂_g_ is a current generator that stands for photogeneration current. (a) Simple recombination model of eq 21 with added series resistance *R*_s_. (b) Standard circuit for halide perovskite frequency techniques.

The circuit in Figure 12a provides a useful picture for the interpretation of the excess apparent lifetime. By eqs 7, 12, and 20 we obtain the identity
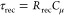
26In the equivalent circuit approach the recombination lifetime is a time constant of the type τ = *RC*. From the impedance spectra, one can obtain the different resistances and capacitor elements, and many types of capacitors are possible in complex devices.^35^ In the model of Figure 12a, the dominant capacitance will prevail. Only if *C*_μ_ > *C*_d_ is the product τ = *RC* interpreted as a recombination lifetime, as indicated in eq 26. Therefore, a main criterion to obtain a recombination lifetime in homogeneous conditions is the clear observation of the chemical capacitance, as indicated in Figure 5b.

In the field of metal halide perovskites, a multitude of measurements of IS and also studies of IMVS and IMPS have been presented, and the knowledge has been summarized recently.^9,48^ An equivalent circuit usually used in these measurements is outlined generally in Figure 12b for any small perturbation measurement, and it reduces to that of Figure 13a for the case of impedance spectroscopy. The circuit shows two arcs in the impedance complex plane (Figure 13b) and two correspondent capacitances. *C*_1_ is a low-frequency capacitance that increases strongly with illumination, while *C*_g_ is a nearly constant high-frequency dielectric (geometric) capacitance,^49^ as indicated in Figures 6a and 13d. Very often a third arc associated with contact layers with an additional capacitance contribution can be observed.^50,51^

**Figure 13:**
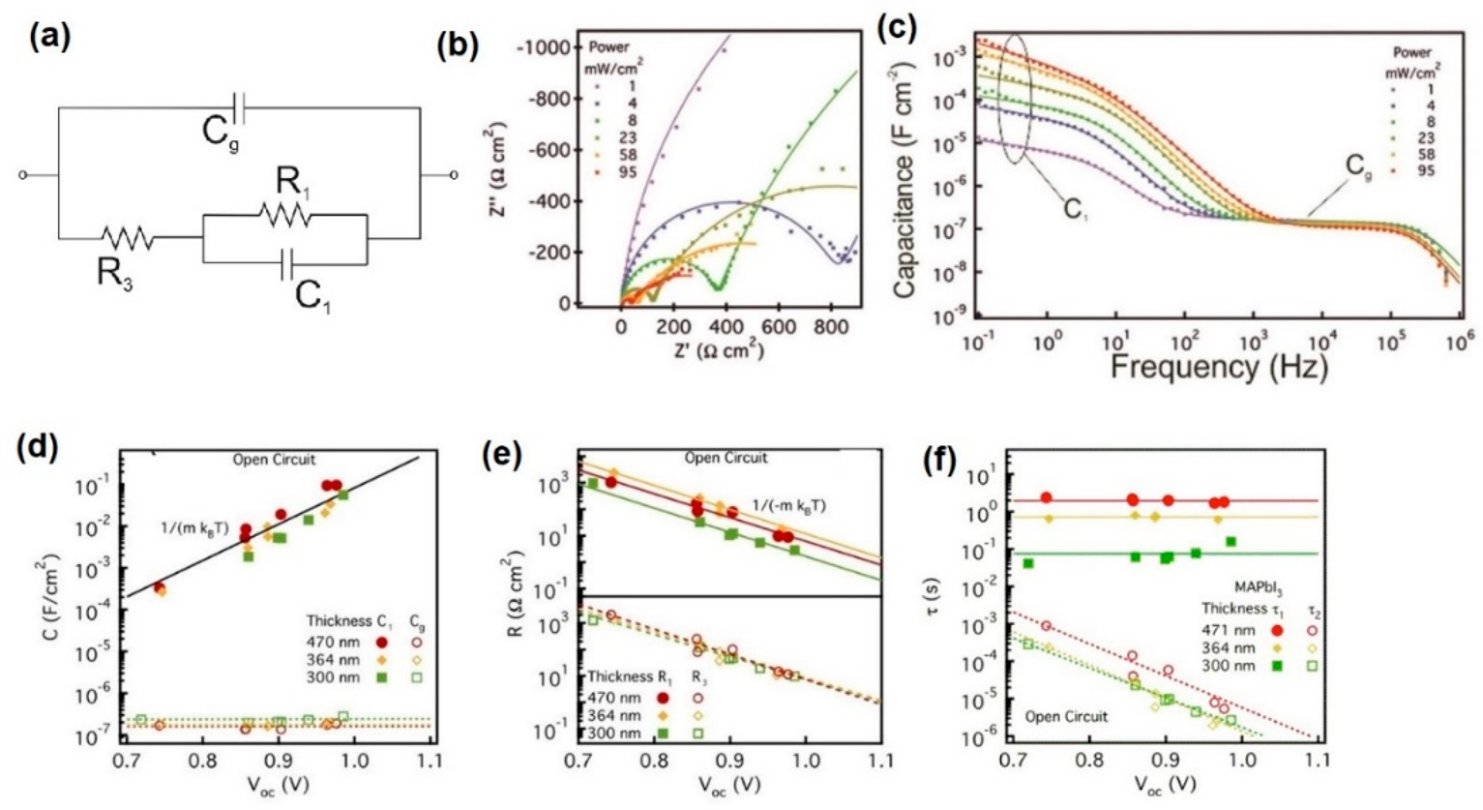
Impedance spectroscopy results of a planar structure FTO/TiO_2_/MAPbI_3_/spiro-OMeTAD/Au solar cell. (a) Equivalent circuit model. (b) Example of complex plane impedance plot measured under short-circuit conditions at different irradiation intensities. Solid lines correspond to fits using the EC of panel a. (c) Example of capacitance spectra corresponding to the conditions in panel b. (d) Capacitances, (e) resistances, and (f) characteristic times under open-circuit conditions. Solid lines (low-frequency arc) and dashed lines (high-frequency arc) correspond to linear fits with *m* approaching 2, in agreement with a second-order recombination. In panel d, *m* = 1.90 ± 0.17, and in panel e, *m* = 1.94 ± 0.08. Reproduced from ref (46). Copyright 2016 American Chemical Society.

In the simplest impedance response of Figure 13a, two resistances are observed that dominate the high-frequency (*R*_3_) and low-frequency (*R*_1_) ranges. The properties of these resistances are shown in Figure 14. Note in Figure 14 that the shunt resistance dominates at low voltages. In some cases these resistances display a similar dependence on illumination and voltage.^46,52^ This is particularly observed for 3D perovskite in the simplest formulations, as shown in Figure 14a for MAPbI_3_-based planar solar cells with four different contacting layers. Both resistances exhibit voltage dependences of the type *R* ∝ e^–*qV*/2*k*_B_*T*^ at high forward potentials. These resistances are interpreted as components of the recombination resistance (eq 20), indicating that similar densities of electrons and holes participate in a second-order carrier recombination mechanism, in agreement with the curves in Figures 8 and 10b at large *V*_oc_ values. However, for multicomponent perovskite absorbing layers shown in Figure 14b the two resistances behave differently, with exponents *m* = 2 and *m* = 1.5, indicating a much more complex situation associated with decoupling of recombination mechanisms.^43^

**Figure 14:**
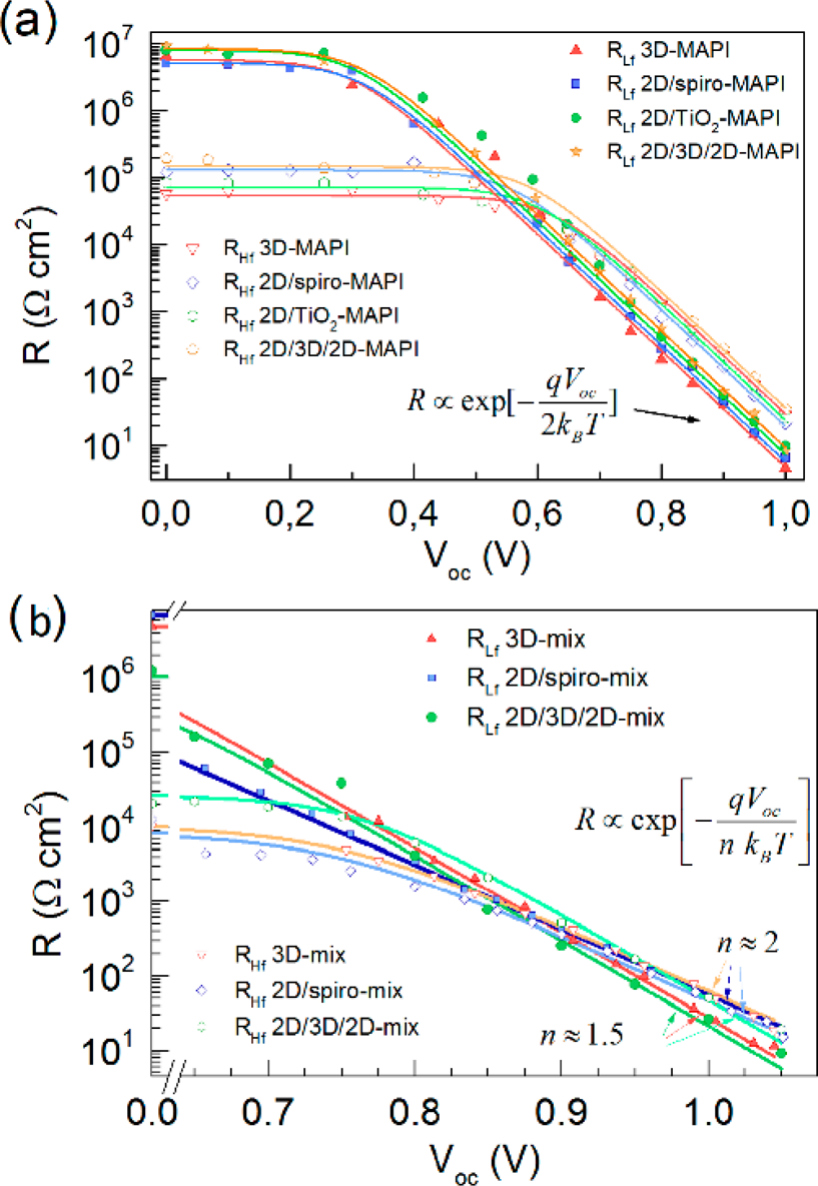
High- and low-frequency resistance as a function of voltage for a variety of solar cells containing different perovskite absorber 3D layers based on (a) CH_3_NH_3_PbI_3_ or (b) mixed Cs_0.1_FA_0.74_MA_0.13_PbI_2.48_Br_0.39_ and a variety of interlayers (2D perovskite thin capping). Adapted with permission from ref (43). Copyright (2018) Elsevier.

The circuit of Figure 12b is not unique. There are other possible connections used by different authors. This question has been reviewed recently.^9,53^ However, the main point to illustrate here is that the circuit in Figure 12a that arises from the recombination model (eq 4) has two capacitances in parallel and gives only one arc in the impedance complex plane. However, the impedance data requires two distinct capacitors that are visible by the presence of different internal resistances. The capacitances are clearly distinguished in the representation as a function of frequency (Figures 6a and 13c). The constant *C*_g_ is neatly separated at high frequencies. However, the issue becomes more complicated because the rise of the capacitance can occur at very high frequency, as shown in Figure 15.

**Figure 15:**
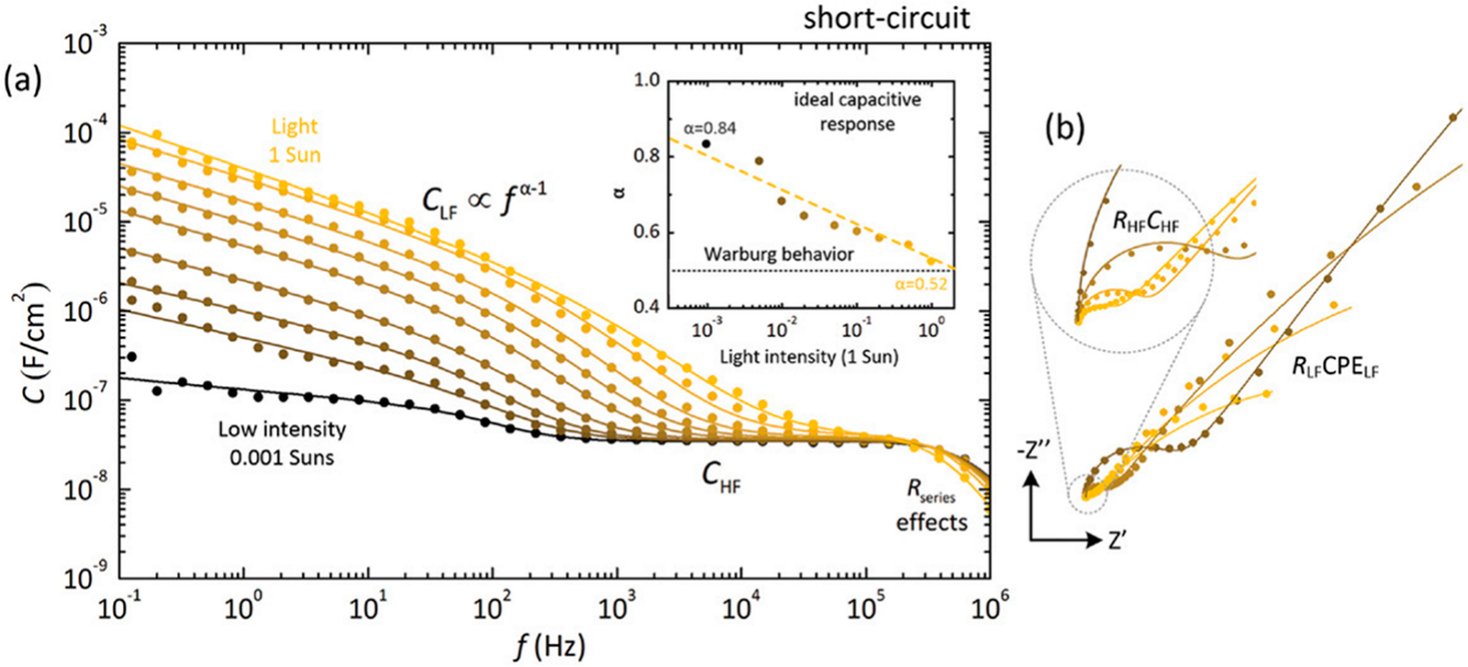
(a) Capacitance spectra and (b) impedance plots, measured under short-circuit conditions (0 V) between 1 MHz and 100 mHz, for 600 nm thick CsFAPbIBr-based solar cells as a function of illumination intensity. Inset of panel a: CPE low-frequency exponents from the nonideal low-frequency relaxation processes, α, versus light intensity. Reproduced with permission from ref (47). Copyright 2022 Wiley-VCH GmbH.

There is a large disparity of the results of IS shown in Figures 13 and 14 and those about measured lifetimes shown previously in Figures 5, 8, and 10. The main problem is that IS does not resolve the chemical capacitance, probably because *C*_μ_ ≪ *C*_d_. It has been shown that in contrast to Si devices, chemical capacitances in halide perovskites are not easily observed,^17^ mainly due to a low DOS value.^18^ In fact, Mora-Seró and co-workers documented the vanishing of the chemical capacitance when increasing the amount of perovskite in the solar cell.^53^ This property relates to the contrast of lifetime and relaxation semiconductors.^54^ On the other hand, the observed resistance dependences on voltage are compatible with recombination mechanisms obtained in the transient methods.

Let us discuss this question in more detail. In Figure 13f we obtain two separate time constants. First, τ_1_ = *R*_1_*C*_1_ is a constant at all voltages because of the exponential increase of *C*_1_ shown in Figure 13d. But τ_1_ is in the range of seconds and cannot correspond to a recombination lifetime. It has been interpreted in terms of a combination of ionic-electronic phenomena.^55^ On the other hand, τ_3_ = *R*_3_*C*_g_ shows an exponential decrease in agreement with Figures 5, 8 and 10, but the values of τ_3_ from IS are much higher than those of the other methods. Furthermore, the high-frequency capacitance of Figure 13c does not show the chemical capacitance, in contrast to Figures 5 and 10. Therefore, τ_3_ cannot be interpreted as a recombination lifetime. Nevertheless, some authors have proposed a correspondence of the different techniques. One example is discussed later.

In summary, there is a general problem for the interpretation of the time constants of IS as a recombination lifetime because there is not a clear correspondence between the model that describes well the IS results (Figure 12b) and that used for the analysis of lifetimes (Figure 12a). This introduces a major problem for the interpretation of how lifetime measurements are affected by capacitive contributions in the *C*_d_ component in eq 18. We have indicated a general dependence in eq 19, because as already mentioned the capacitance contains several components. One can see in Figures 6a, 13c, and 15 that the variable component of the capacitance reaches the high frequencies and can affect the lifetimes measured by time transient methods. Therefore, regarding the observed properties of capacitance as directly measured by IS, the chemical capacitance is not observed, and in addition, it is not straightforward to determine which dielectric capacitance value should be applied in a given decay experiment to correct the distortion of the lifetime. To solve this problem, it seems necessary to provide an equivalent circuit consistent with both time decays and the transfer functions of frequency methods at the same time. A correlation of different methods that was realized for organic^22^ and silicon^56^ solar cells has not been established so far for halide perovskites.

In support of the required correlation of methods that we just mentioned, we recall that by using frequency-modulated illumination as an additional input, two additional methods are obtained that may be correlated to IS: the IMVS and IMPS. In fact, IMVS applied at open circuit is a method used to determine electron lifetimes^57,58^ and the quality of the solar cells.^59^ There have been presented abundant studies of IMPS in halide perovskites.^60−63^

For an equivalent circuit that represents a system as in eq 23 and Figure 12a, operated by three external stimuli (*V*, *I*, and Φ), there are three possible separate output/input transfer functions, by elimination of one variable in each case in eq 23, as indicated in Table 1.^8,64^ The corresponding modes of measurement are shown in Figure 16. An example of the results^51^ of the three methods is shown in Figure 17 for carbon-based perovskite solar cells that consist of a scaffold of mesoporous TiO_2_ and ZrO_2_ layers infiltrated with perovskite and do not require a hole-conducting layer.^65^ Note the negative feature in the real axis of IMPS that will be further discussed in the following. It must be also noted that different convolutions occurring at low frequency can be ascribed to ionic–electronic phenomena, including chemical inductors.^66−68^

**Table 1:** Transfer Function of Small Perturbation Frequency Techniques

method	*Î*	*V̂*	*qΦ̂*_in_	transfer function
IS			0	*Z* = *V̂*/*Î*
IMPS		0		*Q* = *Î*/*qΦ̂*_in_
IMVS	0			*W* = −*V̂*/*qΦ̂*_in_

**Figure 16:**
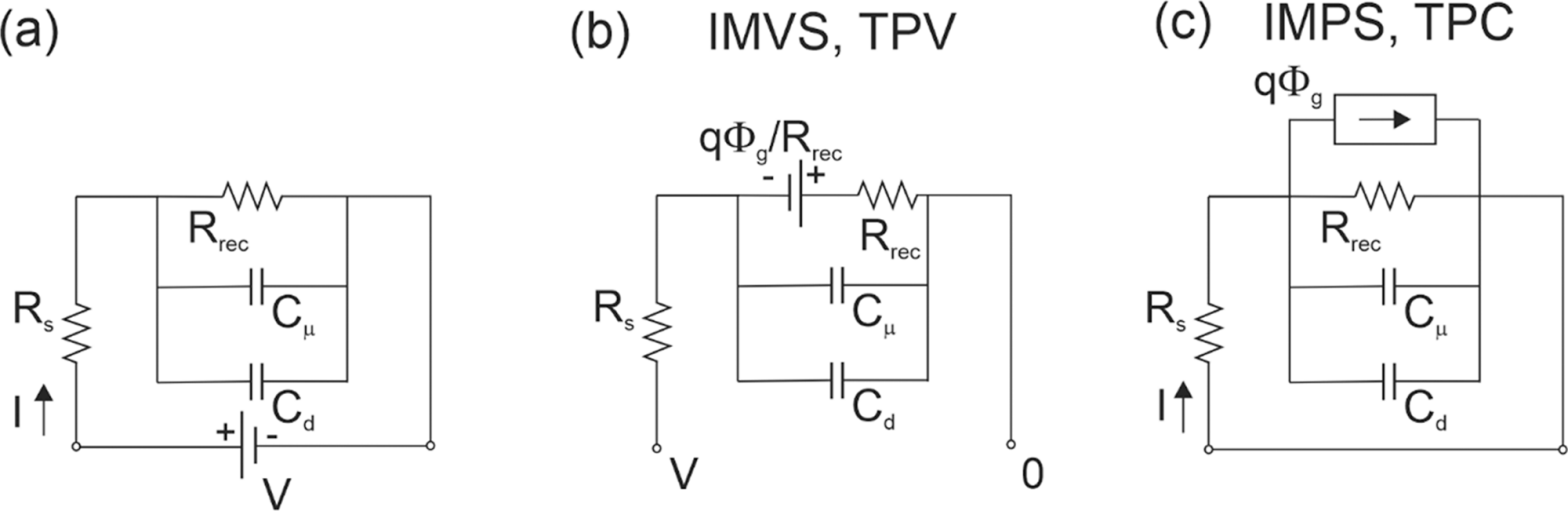
Different setups for the measurement of transfer functions of the model of Figure 11a.

**Figure 17:**
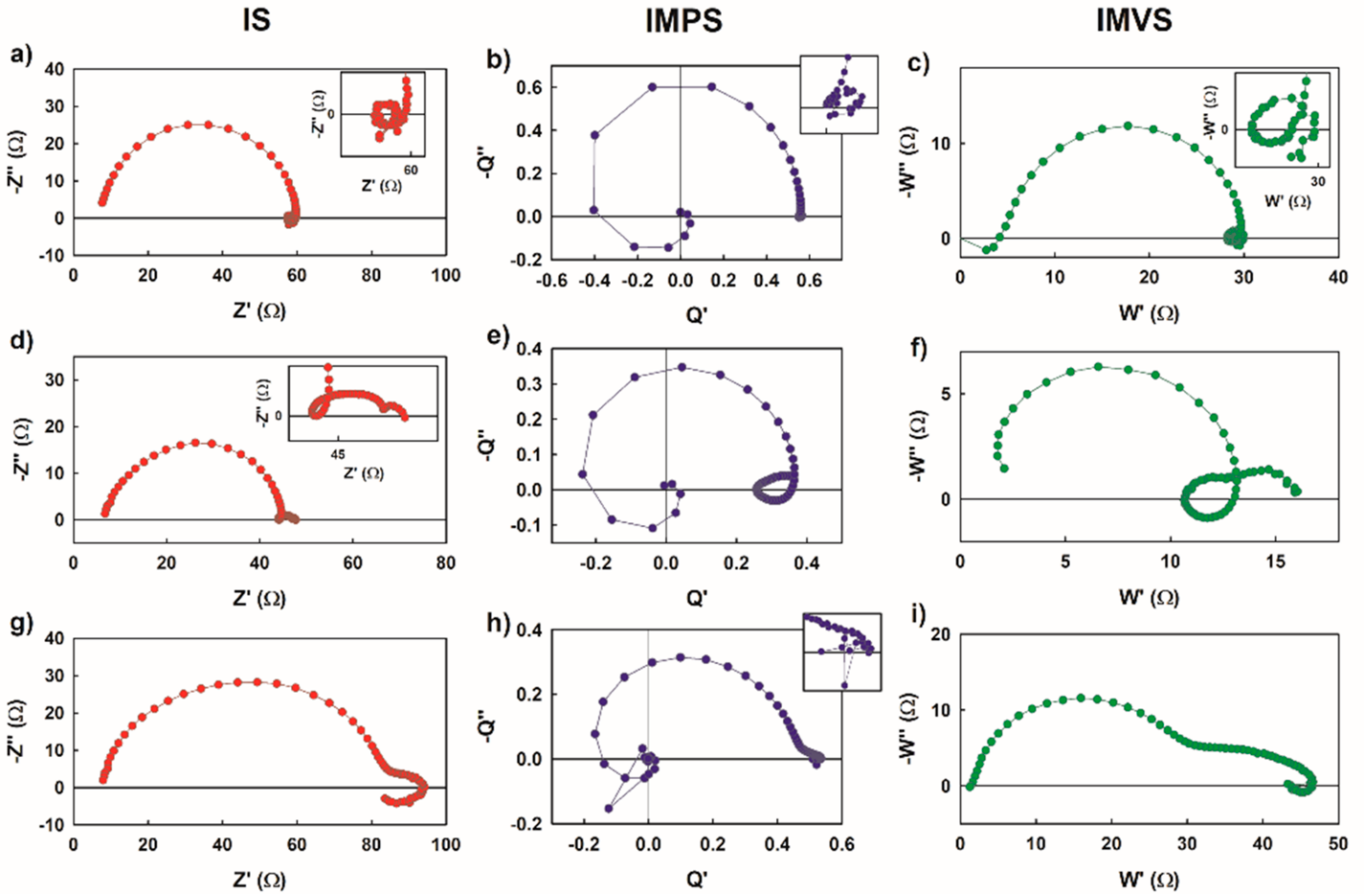
Complex plane plots of the IS (red), IMPS (blue), and IMVS (green) transfer functions (*Z*, *Q*, and *W*, respectively) for a carbon halide perovskite device measured under 0.1 sun illumination, for a frequency range of 1 MHz to 0.01 Hz and at open-circuit voltage. The three rows from top to bottom correspond to cells with regular, double, and triple thicknesses. Adapted from ref (51). Copyright 2020 American Chemical Society.

The identification of the equivalent circuit and its interpretation using independent measurements at the same condition is a major resource for a robust interpretation of the dynamical features of a device.^52,61^ In the case of Figure 16, the spectral shapes should be quite simple. But in practice the situation is complicated by multiple processes, as indicated earlier. It has been shown that an analysis by IMPS reveals distinct features that are lumped in IS.^60^

Consider a general physical model that allows the three independent variations of a device, as in the example of eq 24. As shown by Bertolucci *et al.*,^64^ we obtain a relationship of the type
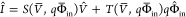
27From Table 1 we can see that eq 27 takes the form
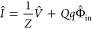
28Therefore, when we arrive at an equation of the type found in eq 27, then we have found the transfer functions because *Z* = *S*^–1^ and *Q* = *T*. As the three transfer functions in Table 1 arise from the small perturbation of a general model function, the triple product rule of the partial derivatives imposes a restriction over the possible values.^8^ The constraint between the three transfer functions is
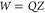
29The relation in eq 29 has been exploited in recent publications.^51,69^ Reference (8) provided the connection between the time and frequency domain for a range of models, including that in Figure 16. Figure 18 shows the correlation between the time constants of IS, TPV, and IMVS of two different halide perovskites solar cells.^29^ The match is excellent in both cases. However, the question of whether this time constant can be interpreted as a recombination lifetime depends on the observation of the chemical capacitance, as noted earlier.

**Figure 18:**
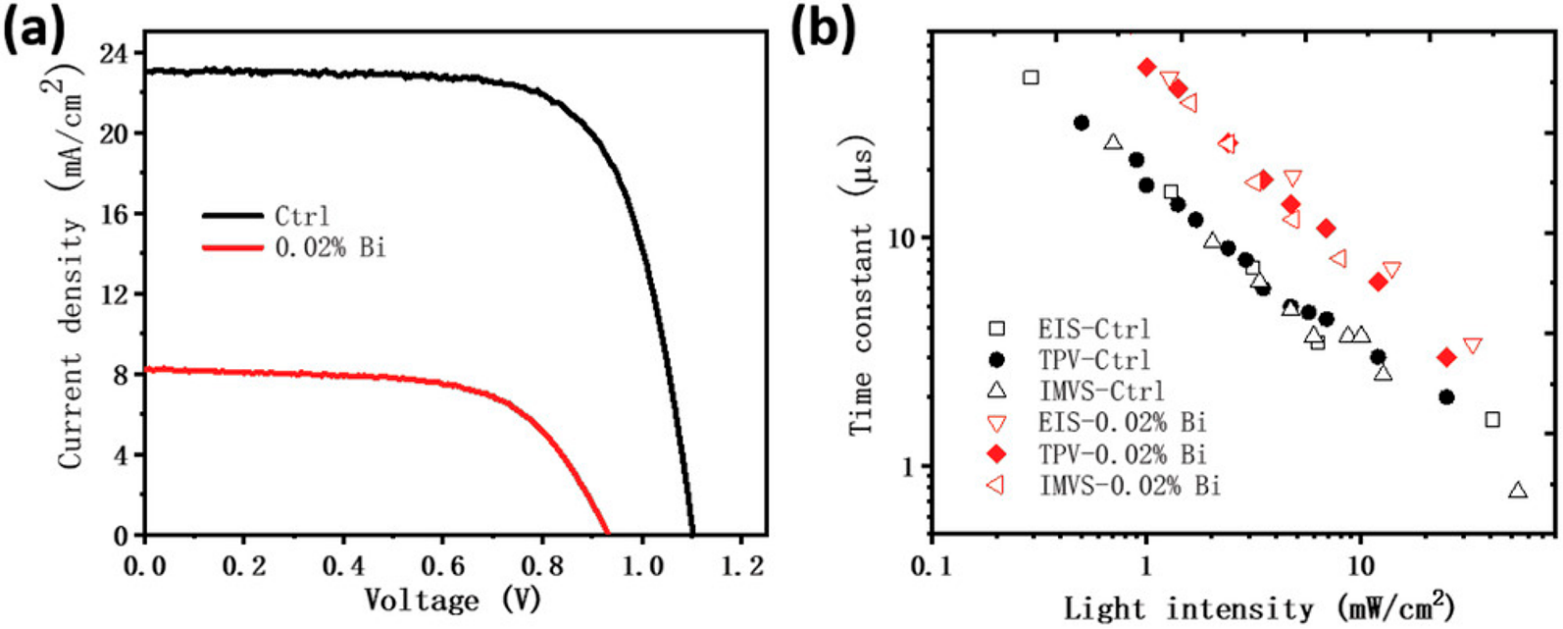
Characterization of solar cells with low (Ctrl) and high defect concentrations (0.02% Bi). (a) *J*–*V* curves of devices measured under simulated solar light (scan rate of −50 mV s^–1^). (b) Small signal perturbation data from TPV, IMVS, and IS measured under white LED light. Reproduced with permission from ref (29). Copyright 2020 The authors of ref (29).

The work on Figure 19 by Pockett *et al.*(70) shows an excellent correlation of nontrivial features in time and frequency domain. The IMVS shown in Figure 18b displays a negative feature curling around the origin. This feature is a transient effect as it is clearly decreasing with time, very likely because of ionic rearrangement at the surface. The TPV signal develops an initial negative spike as shown in Figure 19c. Figure 19d compares the evolution of both negative signals. For IMVS, it is taken as the magnitude of the negative arc crossing the real axis, normalized to the initial value. For TPV, the amplitude of the negative inflection point of the transient response is plotted, again normalized to the initial value. Excellent agreement can be seen between these two different measurements, which provides evidence that the true IMVS response at high frequency has been measured. Further interpretation of negative spikes has been described.^68^

**Figure 19:**
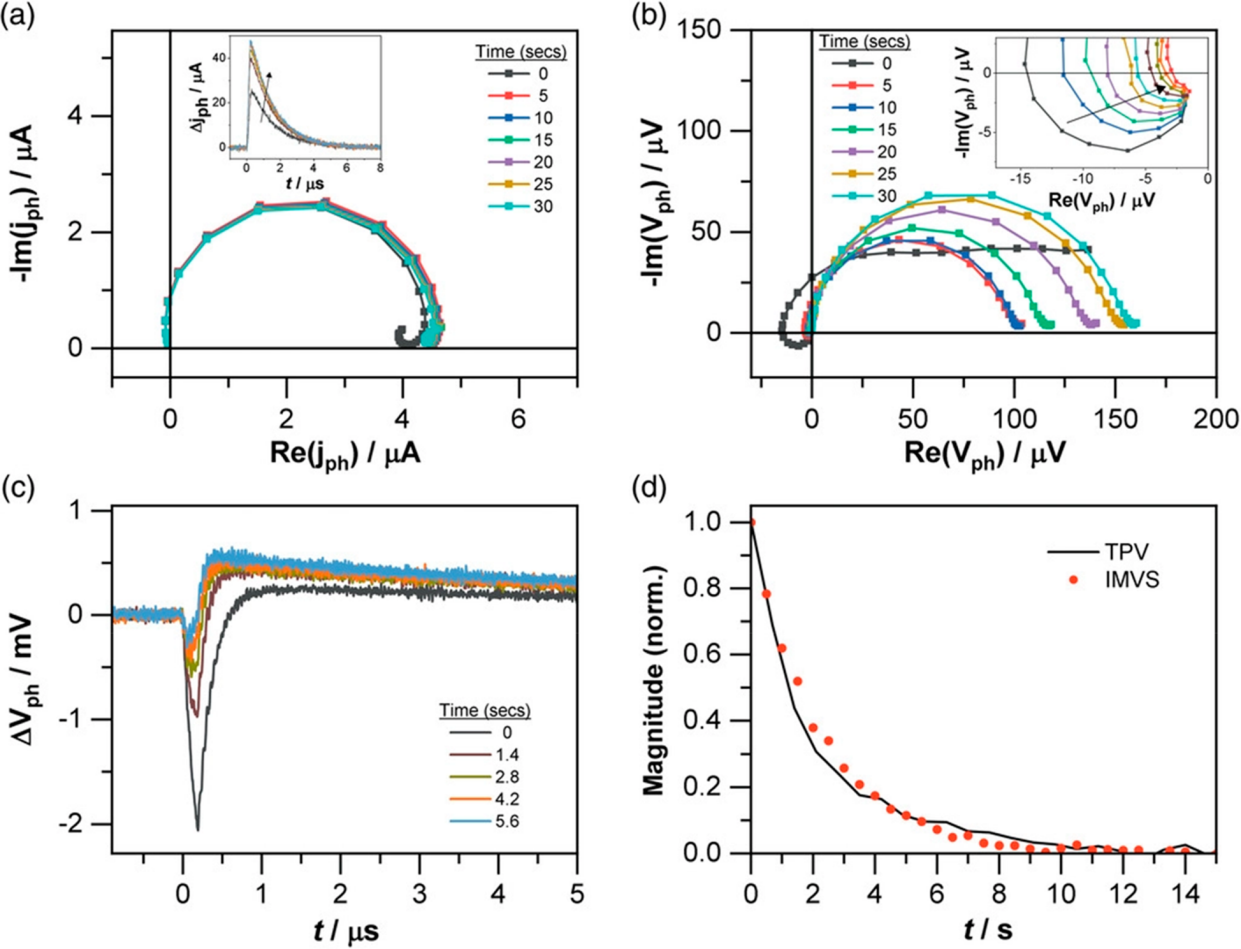
(a) Progression of IMPS response for SnO_2_-based perovskite solar cell at 5 s intervals, showing that no negative photocurrent signal is observed (inset: TPC progression for same SnO_2_ device at 1.4 s intervals, showing that no negative TPC signal is seen even at short times after illumination). (b) Progression of IMVS response for SnO_2_ cell at 5 s intervals (inset: 0.5 s intervals for first 5 s after illumination). (c) TPV response at 1.4 s intervals. (d) Comparison of the relative magnitude of the negative photovoltage signal from IMVS and TPV measurements of SnO_2_ device. Reproduced with permission from ref (70). Copyright 2021 The authors of ref (70).

Recently, it was observed that IMPS spectra show negative values at high frequency, especially occurring in long halide perovskite cells, such as the carbon cell reported in Figure 17b. It was shown^71^ that such a negative value is a feature of the diffusion–recombination model,^73,74^ which can be formulated as
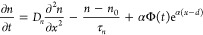
30Here, *D*_*n*_ is the diffusion coefficient, *n*_0_ the equilibrium density under dark conditions, τ_*n*_ the recombination lifetime, *d* the solar cell thickness, and α the light absorption coefficient. A scheme of the model showing the spatial distribution of charge is indicated in Figure 20. By solving the transfer functions, we obtain the following forms according to the three characteristic frequencies indicated in Table 2, namely, ω_rec_, ω_*d*_, and ω_α_.^8,72^ For IS
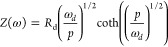
31where *R*_d_ is the diffusion resistance and *p* is defined as
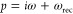
32For IMPS
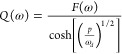
33and for IMVS
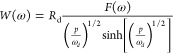
34The transfer functions of IMPS and IMVS have a common factor *F*(ω) that is defined as
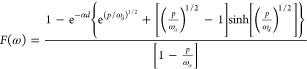
35Note that *Q*_0_, the low-frequency value of IMPS, depends on the series resistance, not included in eq 30, and on the absorptance of the sample.^8,69^

**Figure 20:**
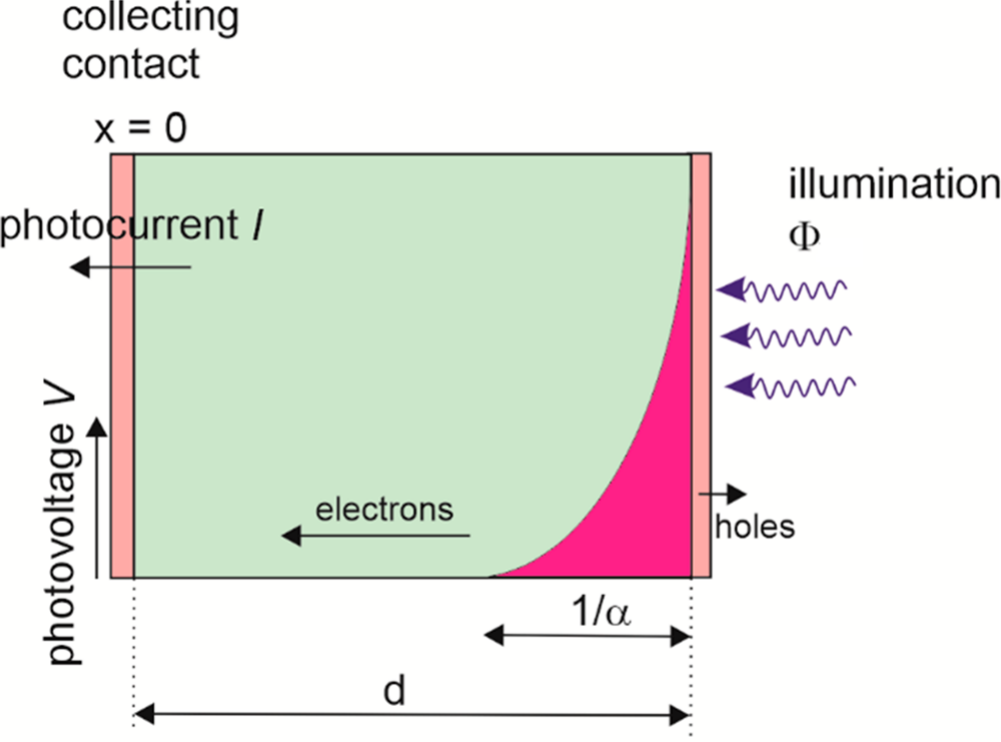
Scheme of the IMPS and IMVS measurement of a solar cell. Here, the illuminated side (*x* = *d*) is the selective contact for holes, and the photogenerated electrons travel by diffusion to the other electrode (*x* = 0) where current and voltage are measured. Reproduced from ref (71). Copyright 2021 American Chemical Society.

**Table 2:** Characteristic Frequencies of the Generation–Diffusion Recombination System and Their Relationships

ω_rec_ = τ_*n*_^–1^	recombination
	diffusion over the cell thickness *d*
ω_α_ = *D*_*n*_α^2^	diffusion over light absorption distance
	diffusion length to film size
	film size to light absorption length
	diffusion length to absorption length

A representation of the spectra is shown in Figure 21. We observe that the negative feature of IMPS and IMVS contains direct information on the recombination lifetime, because ω_rec_ can be identified with good spectral resolution at high frequency, quite apart from the ionic distortions that occur at low frequency as indicated in Figure 17. The method has been shown experimentally to provide the lifetime and diffusion coefficient in different experimental configurations.^71^ However, this method requires that the light penetration distance is short, as indicated in Figure 20. It is complementary to those explained in previous sections, but it comes at the price of inducing highly nonhomegenous conditions of carrier density.

**Figure 21:**
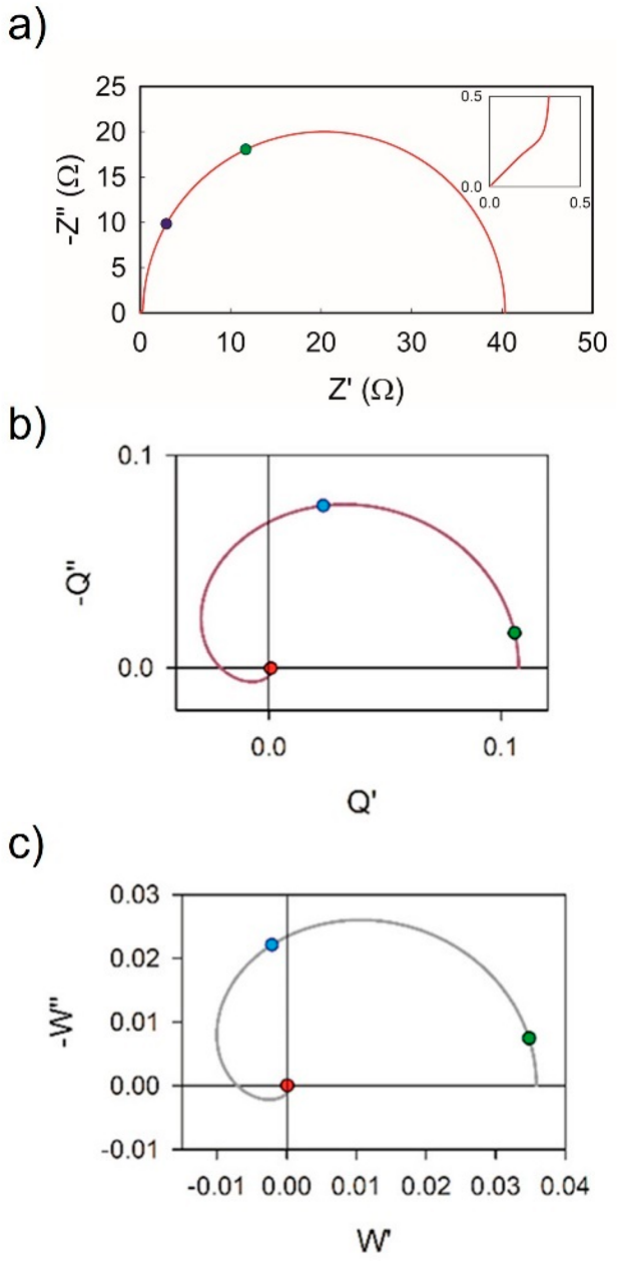
(a) IS, (b) IMPS, and (c) IMVS spectra for typical values of a carbon-based perovskite solar cell. The values of the parameters are *R*_d_ = 1Ω, ω_d_ = 1.2 × 10^5^ s^–1^ (green), ω_α_ = 1 × 10^5^ s^–1^ (red), and ω_rec_ = 5 × 10^4^ s^–1^ (blue). The inset in panel a shows the IS spectrum at high frequency. Reproduced with permission from ref (72). Copyright 2022 The Authors of ref (72).

Figure 22 shows the measurement of the three transfer functions of IS, IMPS, and IMVS for a carbon-based perovskite solar cell.^72^ The experimental spectra in Figure 22a show the shapes of the model in Figure 21. It is noticed that the IMPS and IMVS show the diffusional negative part, while the IS shows only positive values. The reason for this is that the light generation produces information included in the factor of eq 34 that is present in both IMVS and IMPS, but not in IS. The factor disappears when calculating the impedance in eq 31 by the division of eq 29, *Z* = *W*/*Q*.

**Figure 22:**
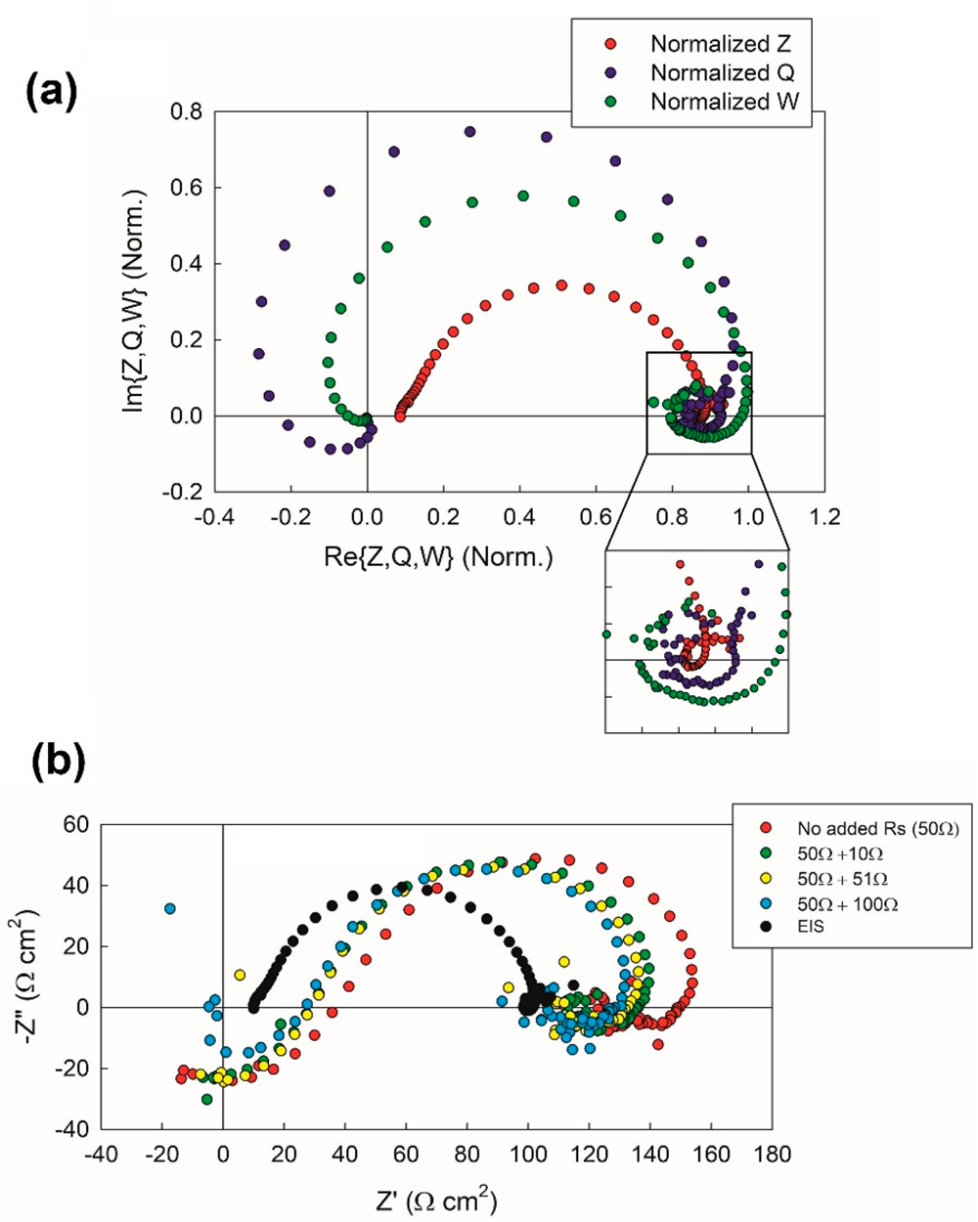
Experimental complex plane plots of the IS, IMPS, and IMVS for a carbon-based perovskite solar cell. Panel a shows the three normalized spectra together. (b) Experimental complex plane plots of the directly measured IS (black points) and the experimental quotient of IMPS and IMVS given by eq 28. Reproduced with permission from ref (72). Copyright 2022 The authors of ref (72).

Figure 22b shows the experimental realization of the division of IMVS and IMPS data. It is confirmed that the negative parts of *Q* and *W* cancel out, leaving only positive values for *Z*. Going back to Figure 17, we note that IMPS contains a real negative part but IMVS does not. This is inconsistent as we have observed in Figure 22, and it has to be attributed to an experimental error of the measurement of *W* at high frequency. Such a defect is resolved in the experimental setup developed by Pockett and co-workers,^70,72^ as shown by the positive answer to consistency tests that has been obtained in Figures 19 and 22.

In summary, it is found that the light-modulated techniques contain information that is not present in IS.

In conclusion, a variety of methods allow measuring the response time in optoelectronic devices with contacts, using a combination of physical signals based on light absorption and emission, voltage, and current. Such response times need a suitable interpretation that we have provided here in terms of *RC* products based on the equivalent circuit model of any small perturbation method over a stabilized stationary state. The identification of the recombination lifetime is based on the criterion that the chemical capacitance and the recombination resistance are clearly observed. In the field of halide perovskites there have been reports of the lifetime dependence on the internal voltage. However, the interpretation of capacitances and recombination resistances is still an issue that requires further investigation. The consistency of different methods (IS, IMVS, IMPS, and TPV) is an important tool to establish the intrinsic dynamic properties. In the case of elementary decay models, all techniques give the answer. We showed that in more complex multicomponent samples or in nonhomogeneous conditions, the different methods are consistent but they may show different pieces of information about the sample behavior.
